# Influence of Anemia on Postoperative Cognitive Function in Patients Undergo Hysteromyoma Surgery

**DOI:** 10.3389/fmolb.2021.786070

**Published:** 2021-11-26

**Authors:** Zhijian You, Lesi Chen, Hongxia Xu, Yidan Huang, Jinglei Wu, Jiaxuan Wu

**Affiliations:** ^1^ Department of Anesthesiology, Liuzhou People’s Hospital, Liuzhou, China; ^2^ Department of Anesthesiology, The Second Affiliated Hospital of Shantou University Medical College, Shantou, China; ^3^ Quality Control Department, Liuzhou People’s Hospital, Liuzhou, China

**Keywords:** postoperative cognitive dysfunction, anemia, uterine fibroid surgery, inflammatory factors, anesthesia

## Abstract

Cognitive dysfunction is a common disease in aging population. This study aims to compare the influence of different degrees of anemia on the cognitive function of patients undergo hysteromyoma surgery. Sixty-one patients aged 18–60 years who underwent uterine fibroid surgery in the Second Affiliated Hospital of Shantou University Medical College from March 2019 to December 2020 were selected for this study. Patients were divided into three groups: group normal (Group N, patients have no anemia), group of mild anemia (Group Mi, patients have mild anemia) and group of moderate anemia (Group Mo, patients had moderate anemia). Combined spinal and epidural anesthesia were administered. Cognitive function tests were performed 1 day before the surgery and repeated at the 5th and 30th days after surgery. Peripheral venous blood samples from patients were collected before the surgery, right after surgery and at the 24th and 72nd hours after surgery. The contents of S-100β, IL-6, TNF-α and IL-1β in serum samples were determined by ELISA. It was found that there were no significant differences in general characteristics of patients among Group N, Group Mi and Group Mo (*p* > 0.05). Nine patients developed postoperative cognitive dysfunction after surgery, and the incidence was 14.75% (9/61). The incidence of postoperative cognitive dysfunction (POCD) was 40% in Group Mo, which was higher than that in Group N and Group Mi. The difference was statistically significant (*p* < 0.05). Inflammatory factors in patients with POCD were higher in post-surgery than before-surgery (*p* < 0.05), while there was no statistical significance in the difference of inflammatory factors of patients without POCD before and after surgery (*p* > 0.05). Taken together, this study suggested that moderate anemia could be a risk factor of POCD in patients undergoing hysteromyoma surgeries. This study will help surgeons developing measures for preventing the occurrence of POCD.

## Introduction

Postoperative Cognitive Dysfunction (POCD) is a concept that was formally introduced in 1988 by the International Study Group on Postoperative Cognitive Dysfunction, a group of 13 medical centers in eight countries (Moller et al., 1998). This concept describes a common central nervous system complication that occurs after anesthesia and surgery, and is characterized by brain dysfunction and cognitive abnormalities that occur within days to months after anesthesia and surgery (Cascella et al., 2018). The incidence of POCD ranges from 8.9 to 46.1% (Androsova et al., 2015). POCD not only increases the incidence of postoperative complications, hospitalization costs, and consumption of scarce medical resources, but also greatly affects patients’ quality of life. The pathogenesis of POCD has not yet been clearly defined, and few study has been conducted to develop clinical treatment options for patients with POCD. Therefore, research on the risk factors and mechanisms of POCD and early intervention in patients with risk factors are important for the prevention of POCD.

Several studies have shown that advanced age is a risk factor of POCD in both cardiac surgeries and non-cardiac major surgeries. In addition, education level, preoperative comorbidities such as cardiovascular disease, preexisting cognitive impairment, anesthesia and surgery are associated with the development of POCD (Rundshagen, 2014). There are different opinions on the effect of anesthesia on POCD. Some studies showed that intraspinal anesthesia has a significantly lower incidence of POCD compared with general anesthesia (Edipoglu and Celik, 2019), while other studies found that neither intraspinal anesthesia nor general anesthesia has a significant effect on the postoperative cognition of patients (Mason et al., 2010). It has been found (Liang et al., 2016) that the incidence of postoperative cognitive dysfunction is significantly higher in patients undergo cardiac surgery than non-cardiac surgery.

Previous studies suggested that the development of POCD may be related to the following mechanisms: 1) Inflammatory response of the nervous system: surgery and anesthesia cause an imbalance between pro-inflammatory and anti-inflammatory substances, resulting in the production of large amounts of inflammatory factors in the peripheral tissues, which enter the central nervous system through the blood-brain barrier, causing an inflammatory response in the central nervous system (Terrando et al., 2010). These inflammatory factors damage neurons and synapses by promoting oxidative stress, affecting the synaptic transmission function and causing POCD in patients (Tang et al., 2017; Li et al., 2018; Netto et al., 2018; Lin et al., 2019). 2) Abnormal protein function: β-amyloid, which is closely related to neurological impairment, is often recognized as an important marker of postoperative cognitive function (Pannee et al., 2016; Zhang et al., 2017; Xu et al., 2019), and was found in animal experiments by PLUTA (Pluta et al., 2020). And another protein, Tau protein, was also significantly correlated with neurological impairment (Lee et al., 2017). It was found that increased phosphorylated Tau protein in neuronal cells led to structural damage and synaptic loss, resulting in neuronal dysfunction and postoperative cognitive impairment. 3) Cholinergic system: It has been suggested (He et al., 2012; Egea et al., 2015; Talita et al., 2016) that hypofunction of the cholinergic system may cause POCD, for the cholinergic pathway can reduce the systemic inflammatory response by inhibiting the release of pro-inflammatory factors. If the cholinergic system becomes dysfunctional and pro-inflammatory factors are released, they might cause an imbalance between pro- and anti-inflammatory substances. It is possible that the patient may experience cognitive impairment after the procedure.

Uterine fibroids are benign tumors of the reproductive system. Clinically, patients with uterine fibroids often suffer from varying degrees of anemia, a chronic loss of blood that is classified as microcytic hypochromic anemia, caused by the long-term loss of red blood cells and the inability to reabsorb iron from them. It has been found that patients with iron deficiency anemia have significant cognitive impairment, and the degree of cognitive impairment is related to the duration and condition of iron deficiency anemia (Chan et al., 2001; Wang et al., 2017). However, the specific mechanism of POCD caused by anemia is not clear. Anemia causes hypoxia in tissues and organs, and brain tissues are sensitive to hypoxia. Nerve cell damage may occur when brain tissues are hypoxic (Nielsen, 2014; Colak et al., 2015). In addition, most of the enzymes involved in cellular redox contain iron or are iron-dependent, and the long-term loss of red blood cells in patients with anemia prevents the reabsorption of iron from red blood cells, causing a decrease in the activity of these iron-containing or iron-dependent redox enzymes. Decreased enzyme activity affects neurological function of the brain, resulting in mental and behavioral changes of patients (Yang et al., 2011). On the other hand, it has also been found that patients with chronic blood loss (anemia) are easier to get infections. In a review of 11 years of hospitalized patients with peptic ulcers combined with chronic hemorrhagic anemia, Cai et al. found that anemia was a risk factor for infection, and the degree of anemia was positively associated with the incidence of infection (Cai et al., 2005). In patients with infections, elevated peripheral blood inflammatory factors were measured and were used as predictors of the early course of infection (Medina Fernández et al., 2015; Backes et al., 2018).

In a study by Cestaric, the level of serum interleukin (IL)-6 was found to be higher in elderly patients with cognitive impairment and there was a correlation between elevated IL-10 and IL-12 and amyloid deposition (Dong et al., 2012; Xie and Xu, 2013). Both the inflammatory response to infection and the chronic non-infectious inflammatory response can lead to permanent cognitive complications (Eder, 2010; Marcantonio, 2017). A study of patients with severe infections found that they were much more likely to have altered mental status than patients without severe infections. Also, the increased EEG low-frequency activity and hippocampal atrophy in patients with severe infections and long-term cognitive dysfunction suggest that severe infections can lead to substantial brain damage.

In this study, we examine whether chronic anemia in patients with uterine fibroids, which is associated with varying degrees of anemia, contributes to the development of POCD. In addition, we evaluate the association of POCD with inflammatory factors. We grouped patients with different degrees of anemia according to the clinical classification of anemia, and the cognitive function of patients with different degrees of anemia after uterine fibroid surgery was investigated. The results of the study will help prevention and treatment of postoperative cognitive dysfunction.

## Materials and Methods

### Patient Participants and Grouping

Following the approval of the Faculty Ethics Committee of the Second Affiliated Hospital of Shantou University Medical School, 61 patients who underwent elective hysteromyoma resection were recruited in this study. Patients submitted their inform consents. They aged 18–60 years old and have ASA I-II physical status. The following patients were excluded from the study: 1) Patients with limited or low literacy (illiterate), unable to communicate without barriers, unable to cooperate with the examination; 2) patients known to have neurological or psychiatric diseases affecting the central nervous system (CNS) and cognitive functions; 3) patients who were taking medications affecting CNS; 4) patients who have undergone previous cognitive function tests; 5) patients had any adverse events such as drug allergies; 5) patients who met the diagnostic criteria of anxiety or depression after assessment with the Self- Assessment Scale for Anxiety and the Self-Assessment Scale for Depression; 6) patients who underwent a second operation or were admitted to ICU after surgery; and 7) patients with alcohol or any substance addictions. Patients who have not completed all the steps and procedures shown in the experimental protocol, such as preoperative and postoperative cognitive function assessment and peripheral venous blood collection were also excluded from the study.

Patients included in the study were divided into three groups according to the preoperative hemoglobin level of the patients, including normal group (Group N, patients who had preoperative hemoglobin levels over 110 g/L), mild anemia group (Group Mi, patients who had preoperative hemoglobin level between 90 and 110 g/L) and moderate anemia group (Group Mo, patients who had preoperative hemoglobin levels between 60–90 g/L). In addition, 15 healthy volunteers without surgery were recruited in the community as a non-surgical control group in order to eliminate the learning effect of the experimental group in the cognitive function test.

### Preparation of Surgery

Peripheral vascular access was established in patients. The electrocardiogram, peripheral oxygen saturation (SpO2), and non-invasive arterial pressure monitor were performed after patients were taken to the operating room. None of the patients were pre-medicated. After anesthesia was initiated in the patients, oxygen was administered using a face mask or nasal cannula with low oxygen flow (3.0 L/min). Skin disinfection for patients was performed with the antiseptic solution. The epidural cavity was penetrated between the L3-L4 with the epidural puncture needle from mid-line, and then the atraumatic spinal needle was punctured. After the free, clean cerebrospinal fluid flow was observed, ropivacaine was administered for 10–15 s according to the body weight of patients. The epidural catheter was placed into the epidural cavity through the epidural puncture needle. The first epidural dose was 5 ml of 3% chloroprocaine, which was intermittently injected from the epidural catheter at intervals depending on the patient’s level of anesthesia and the length of the procedure, with an additional 5–10 ml of 3% chloroprocaine. Ketorolac at the concentration of 0.5 mg/kg was intravenously administered 20–30 min before the completion of skin closure, and the patient was given a self-administered intravenous analgesia pump (Analgesia pump formulation: bupropion 5 mg + flurbiprofen ester 100 mg + toltestrone 10 mg + 0.9% NaCl solution = 100 ml). Patients’ visual analogue scale (VAS) scores were assessed at the ward at days 1, 3 and 5 after surgery. If patients had a VAS score >3, intramuscular tramadol 50–100 mg was administered to maintain a VAS ≤3.

### Evaluating Patients’ Cognitive Function

To determine the level of the cognitive functions of patients, cognitive function test was performed 1 day before the surgery, 5 days and 1 month after the surgery. Cognitive function tests consist of mini-mental state examination (MMSE), Hopkins Verbal Learning Test (HVLT), Trail Making Test A, B (TMT-A, B), Stroop Color Word Interference Test (SCWIT) and Verbal Fluency test (VF). To exclude enrolled patients who met the diagnostic criteria for anxiety and depression, the Zung’s Anxiety Self- Rating Scale and the Depression Self-Rating Scale were used before and after the surgery.

Peripheral venous blood was collected from the upper extremities of patients the day before surgery, right after surgery and 24 and 72 h after surgery. The blood was centrifuged for 10 min with a high-speed centrifuge set at 3,000 r/min and the serum was extracted and stored in a refrigerator at −80°C. The serum specimens were detected by enzyme-linked immunosorbent assay (ELISA) for S 100β, IL-1β, TNF-α, and IL-6 within 1 month.

### The Blind of Surgeon, Sample Collector, and Sample Analyzer

In the experiment, we simply informed the surgeons in the gynecology department that we would perform the experiment on patients who met the requirements of the experiment, without specifically informing them of the patient’s information. The same physician was assigned to the patient for all three preoperative and postoperative assessments of cognitive function, and this physician knew only the patients’ name and hospitalization number for verification and had no other information of the patients. Venous blood samples were stored after centrifugation in numbered centrifuge tubes, each number corresponding to a particular time period of a particular patient’s blood. The specific meaning of the numbers was not known to the person performing the ELISA and data analysis.

### Statistical Analysis

The measurement data were presented as mean ± SD, and the count data were presented as number of cases or percentage (%). We used SPSS software for data analysis. *t* test was used to compare the measurement data. And the count data were compared by chi-square test. A value of *p* < 0.05 was accepted as statistically significant.

## Results

### General Characteristics of Patients

No subjects were found to meet the diagnostic criteria of anxiety or depressive status. All the patients involved were female with ASA classification of I to II. None of the patients had a history of smoking or alcohol abuse. In addition, none of them had any preoperative comorbidity of severe hypertension, arrhythmias, or other underlying diseases. There was no statistically significant difference among three groups in terms of age, BMI, education, intraoperative bleeding, surgical time and anesthesia time (*p* > 0.05) (Table 1).

**TABLE 1:** Demographic characteristics of patients of normal group, mild anemia group and moderate anemia group (± s).

	Group	Group	Group
	N (*n* = 25)	Mi (*n* = 21)	Mo (*n* = 15)
Age (year)	42.6 ± 6.3	39.8 ± 5.1	43.4 ± 7.9
BMI(kg/m2)	21.5 ± 2.1	21.8 ± 2.6	20.2 ± 3.5
Education level (year)	7.4 ± 2.2	7.0 ± 2.5	8.5 ± 3.8
≤6 years (%)	32	35	30
6–9 years (%)	56	55	30
>9 years (%)	12	10	40
Intraoperative bleeding (ml)	65.0 ± 15.8	54.0 ± 20.4	60.0 ± 12.5
Anesthesia time (min)	124.0 ± 12.8	122.0 ± 15.4	119.0 ± 15.1
Surgical time (min)	121.0 ± 15.6	117.0 ± 16.4	115.0 ± 13.5

### The Postoperative Cognitive Function of Patients

The intra-group comparison of the cognitive function of patients was performed. The postoperative cognitive function tests were performed in each group of patients the day before surgery, the 5th and 30th day after surgery. It was found that the scores of HVLT- I and HVLT-D were decreased, and the time spent on TMT-B, SCWIT C and SCWIT C-B were increased in patients after surgery compared with that before surgery in the Group Mo. The difference between the scores after surgery and before surgery was statistically significant (*p* < 0.05). The remaining three test scores were not significantly different preoperatively and postoperatively. In the patients in Group N and Group Mi, the MMSE, HVLT-I, HVLT-D, and VF scores at postoperative 5th and 30th days increased, and the time spent decreased compared to those before surgeries, which may be due to the repetitive performance of the tests.

In between groups comparison, the preoperative cognitive function test scores showed that the Group Mo had lower scores (or longer time spent) than the Group N and Group Mi on HVLT-I, VF, SCWITC, and SCWITC-B, and the differences were statistically significant (*p* < 0.05). There was no statistically significant difference between Group N and Group Mi. The remaining four tests were not significantly different between two groups in a pairwise comparison. In the postoperative cognitive function tests, except for MMSE, TMT-A and VF, the scores (or time spent) of the remaining five tests in Group Mo decreased significantly (or longer) than those in Group N and Group Mi at postoperative 5th and 30th days, with statistically significant differences (*p* < 0.05). Group N and Group Mi had no statistically significant difference in preoperative and postoperative scores for each test.

### The POCD Patients

Sixty-one patients completed preoperative and postoperative cognitive function tests. Among them, nine patients developed POCD, one from Group N, two from Group Mi and six from Group Mo. Using the chi-square test for paired enumeration data, it was found that the difference in the incidence of POCD between Group Mo and Group N was statistically significant (*p* < 0.05), while the difference in the incidence of POCD between the Group Mi and Group N was not statistically significant (*p* > 0.05) (Table 2).

**TABLE 2:** Comparison of the incidence of POCD in each group.

Groups POCD	Non-POCD	Incidence
Group Surgery (*n* = 61) 9	52	14.75%
Group N (*n* = 25) 1	24	4.00%
Group Mi (*n* = 21) 2	19	9.52%
Group Mo (*n* = 15) 6	9	40.00%^*#^
Compared to Group N,^*^ *p* < 0.05	compared to Group	Mi, ^#^ *p* < 0.05

### The Association of POCD and Inflammatory Factors

**FIGURE 1:**
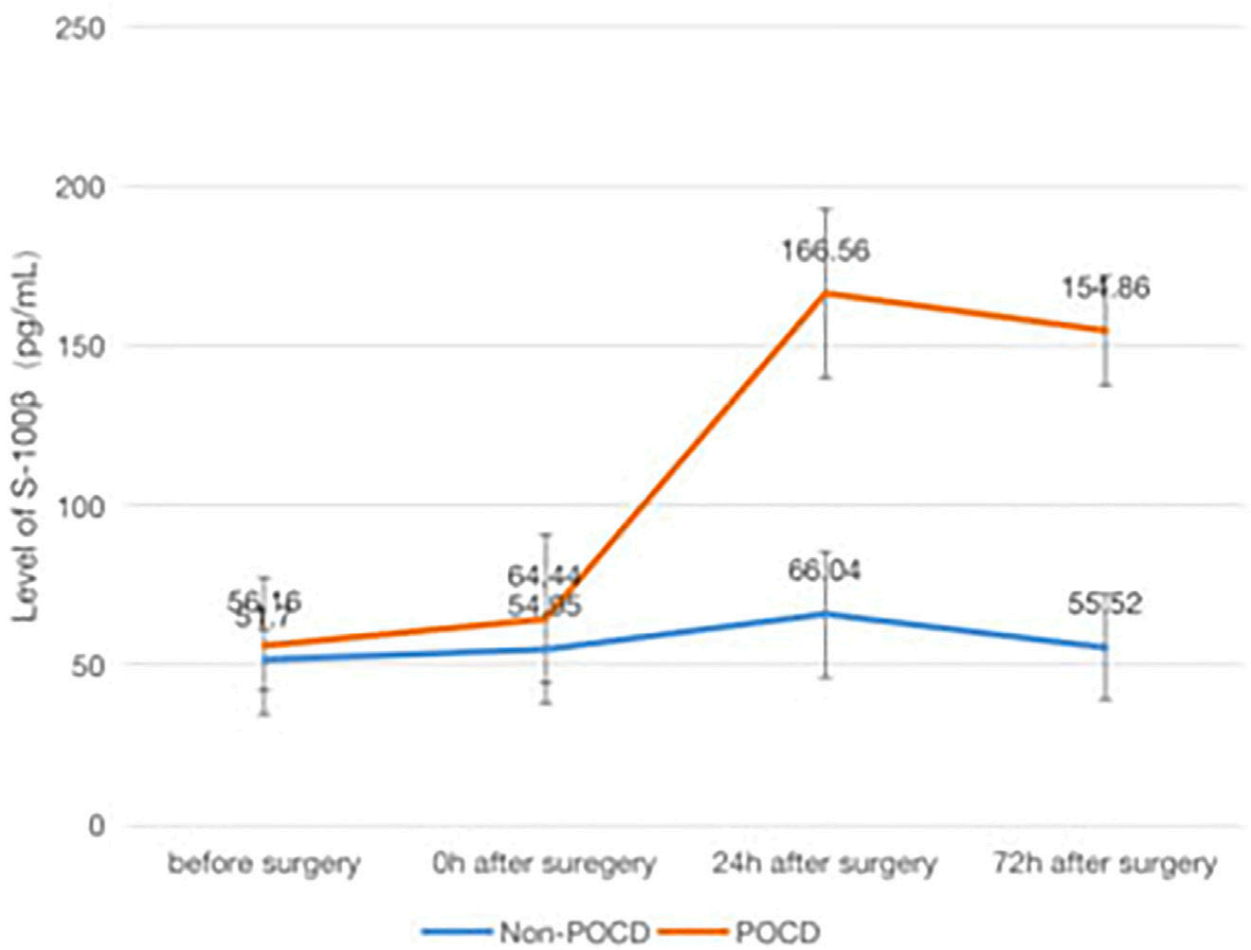
Comparison of serum concentrations of S-100β (pg/ml) between non-POCD and POCD groups before and after surgery丨There was no significant difference in the preoperative levels of S-100β concentration in the Non-POCD group compared with postoperative levels. There was no significant difference in the preoperative levels of S-100β concentration in the Non-POCD group compared with POCD group. Compared with preoperative levels, the levels at 24th and 72nd hours after surgeries in the POCD group increased with statistically significance. The differences in the levels of S-100β concentration between patients in the POCD and Non-POCD groups at the same time points postoperatively were statistically significant. Values are mean ± SD. *n* = 61. *p < 0.05 vs. the preoperative level of S-100β; #*p* < 0.05 vs. the POCD group.

**FIGURE 2:**
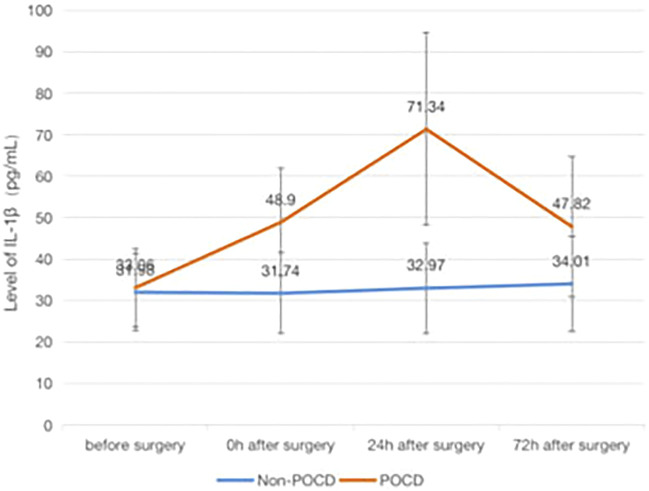
Comparison of serum concentrations of IL-1β (pg/ml) between Non-POCD and POCD groups before and after surgery丨There was no significant difference in the preoperative levels of IL-1β concentration in the Non-POCD group compared with postoperative levels. There was no significant difference in the preoperative levels of IL-1β concentration in the Non-POCD group compared with POCD group. Compared with preoperative levels, the levels at 24th and 72nd hours after surgeries in the POCD group increased with statistically significance. The differences in the levels of IL-1β concentration between patients in the POCD and Non-POCD groups at the same time points postoperatively were statistically significant. Values are mean ± SD. *n* = 61. *p < 0.05 vs. the preoperative level of S-100β; #*p* < 0.05 vs. the POCD group.

**FIGURE 3:**
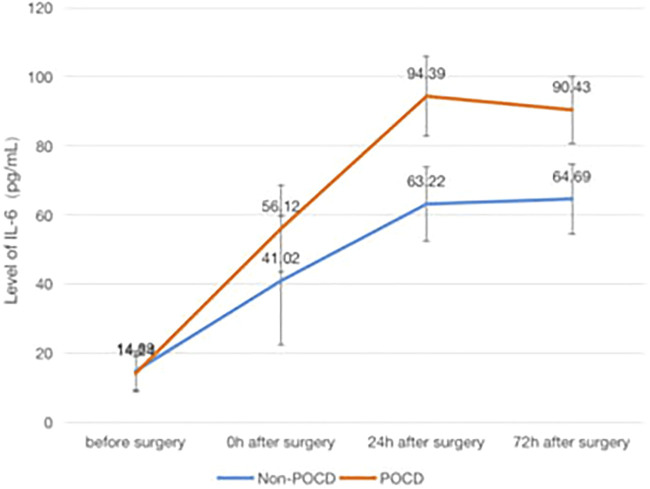
Comparison of serum concentrations of IL-6 (pg/ml) between Non-POCD and POCD groups before and after surgery丨There was no significant difference in the preoperative levels of IL-6 concentration in the Non-POCD group compared with postoperative levels. There was no significant difference in the preoperative levels of IL-6 concentration in the Non-POCD group compared with POCD group. Compared with preoperative levels, the levels at 24th and 72nd hours after surgeries in the POCD group increased with statistically significance. The differences in the levels of IL-6 concentration between patients in the POCD and Non-POCD groups at the same time points postoperatively were statistically significant. Values are mean ± SD. *n* = 61. *p < 0.05 vs. the preoperative level of S-100β; #*p* < 0.05 vs. the POCD group.

**FIGURE 4:**
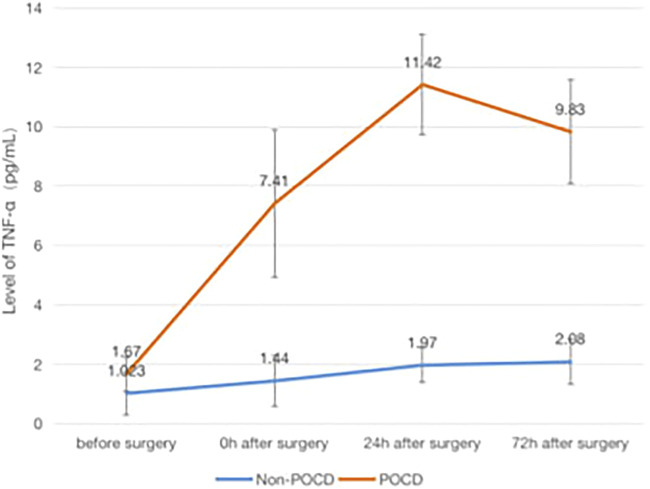
Comparison of serum concentrations of TNF-α (pg/ml) Between Non-POCD and POCD groups before and after surgery丨There was no significant difference in the preoperative levels of TNF-α concentration in the Non-POCD group compared with postoperative levels. There was no significant difference in the preoperative levels of TNF-α concentration in the Non-POCD group compared with POCD group. Compared with preoperative levels, the levels at 24th and 72nd hours after surgeries in the POCD group increased with statistically significance. The differences in the levels of TNF-α concentration between patients in the POCD and Non-POCD groups at the same time points postoperatively were statistically significant. Values are mean ± SD. *n* = 61. *p < 0.05 vs. the preoperative level of S-100β; #*p* < 0.05 vs. the POCD group.

Patients who developed POCD were categorized into the Group POCD, and other patients who did not develop POCD were categorized into the Group Non-POCD. The serum was obtained by centrifugation of peripheral venous blood specimens collected 1 day before surgery, at the end of surgery, at the 24th and 72nd hours after surgeries, and stored in a refrigerator at −80°C. The concentrations of S-100β, IL-1β, TNF-α and IL-6 in the serum were determined by using enzyme-linked immunosorbent assay (ELISA). It was found that the levels of S-100β, IL-1β, IL-6 and TNF-α in the serum of patients in both groups were increased at the 24th and 72nd hours after surgeries compared to those before surgeries, and the increase in the levels of S-100β, IL-1β, IL-6 and TNF-α of patients in the POCD group postoperatively compared to preoperatively was statistically significant (*p* < 0.05), while the difference in the preoperative and postoperative levels of each inflammatory factor concentration in the Non-POCD group was not statistically significant (*p* > 0.05). The differences in the levels of each inflammatory factor concentration between patients in the POCD and Non-POCD groups preoperatively were not statistically significant (*p* > 0.05), and the differences in the levels of each inflammatory factor concentration between patients in the POCD and Non-POCD groups at the same time points postoperatively were statistically significant (*p* < 0.05) (Figures 1–4).

**FIGURE 5:**
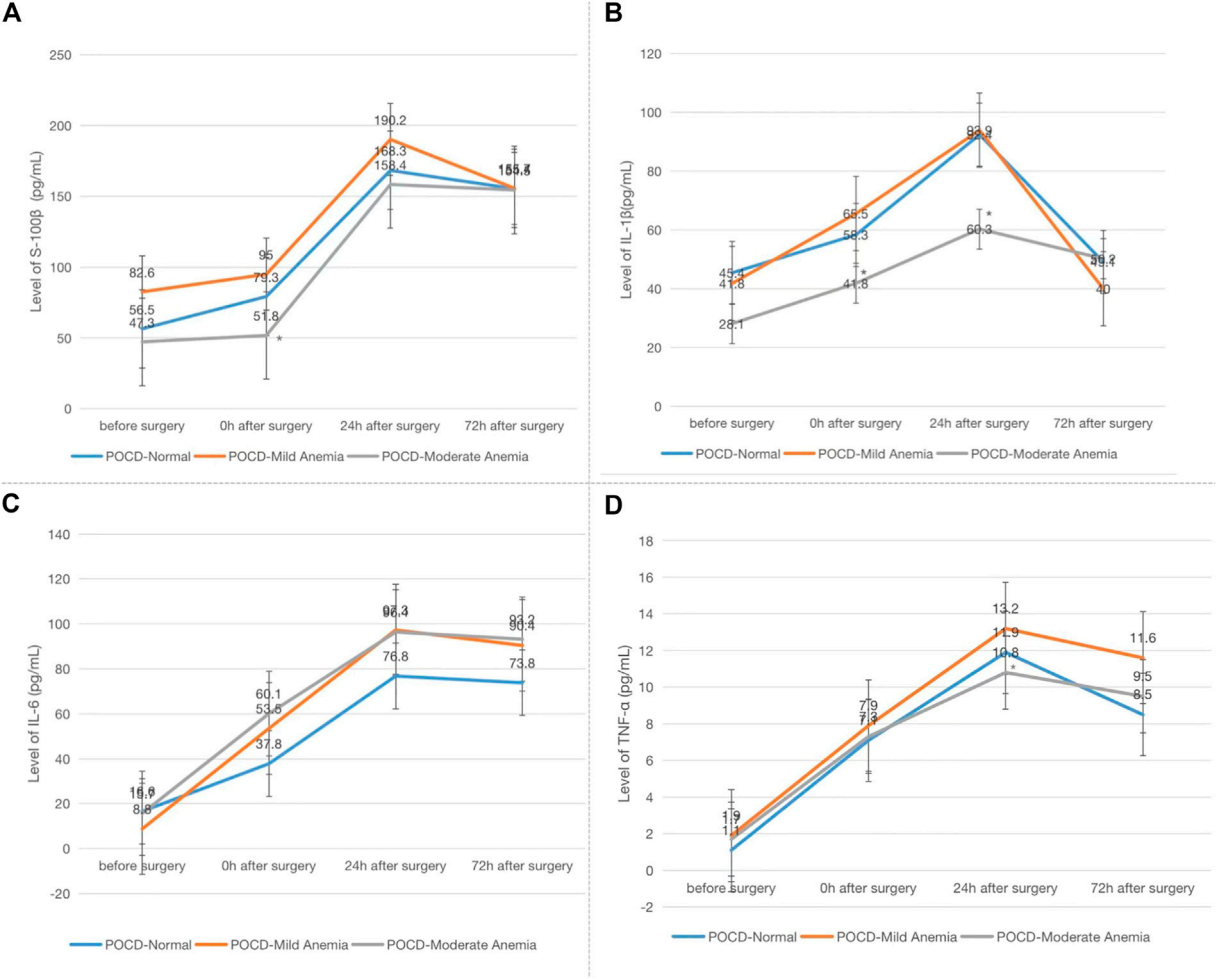
Comparison of serum concentrations of S-100β, IL-1β, IL-6 and TNF-α (pg/ml) among patients with POCD who had no anemia, mild anemia or moderate anemia at four time points. Note: **(A)** S-100β; **(B)** IL-1β; **(C)** IL-6; **(D)** TNF-α. Compared with patients had no anemia or mild anemia in POCD group, *p < 0.05.

By independent sample *t*-test analysis, we found that in patients with POCD who had no anemia or had mild anemia, the concentration levels of S-100β at the end of surgery, IL-1β at the end of surgery and 24 h after surgery, and TNF-α at 24 h after surgery were statistically significant compared with those with moderate anemia (*p* < 0.05) (Figure 5).

## Discussion

POCD is a neurodegenerative condition with impairment of memory, executive and information processing abilities, and personality changes that occurs in the months following surgery and anesthesia. The manifestations of POCD range from mild memory loss to the inability to concentrate or process information received by the brain, all of them may have a significant impact on the patient’s quality of life. Personal factors such as the patient’s educational level, age, gender, preoperative comorbidities, and the presence of preoperative cognitive impairment (Salthouse, 2010; Cao et al., 2014; Wang et al., 2019; Cui et al., 2020; Kiabi et al., 2020), the choice of anesthetic drugs and anesthesia, the mode of surgery, and the length of surgery are all risk factors associated with the development of POCD. In this study, the selected patients have no significant differences in general characteristics, and their anesthesia ASA classification were all ensured to be grade I ∼ II. Patients with combined cardiovascular and cerebrovascular systemic diseases, preoperative cognitive impairment and history of alcohol consumption in the perioperative period were excluded. According to the different anesthesia methods and preoperative anemia, this experiment was divided into four groups. The duration of surgical anesthesia in each case was all less than 3h, and postoperative analgesia ensured that patients’ VAS scores were less than 3. Therefore, the study is comparable under this premise.

There is no clear and uniform diagnostic criterion for POCD, and the currently most widely used one relies on the objective neuropsychological scales to assess the cognitive function of patients before and after surgery. There are several basic requirements for the selection of scales to help assess cognitive function: Firstly, the test should not take too long or consist too many test scales. However, to assess all aspects of cognitive function, it is not enough to rely on a single scale alone. Multiple test scales that can detect all aspects of cognitive function are needed; but if there are too many test scales, the time it takes will increase accordingly. Therefore, the test duration is generally recommended between 30 min and 1 h. Secondly, the test scale is required to be sensitive, and if the ceiling of the test is too low, a capping effect may occur, which means that most normal people will get a score close to a perfect result in the test, thus making it difficult to detect the slight decline in cognitive ability that occurs in POCD patients after surgery. Simple Intelligence Mental State Examination Scale is a scale that usually has a low sensitivity, which may result in an increased false-negative rate of test results, and is only suitable for screening patients with a combination of a higher degree of preoperative functional cognitive impairment. Thirdly, the cognitive function scale should be repeatable in order to satisfy multiple preoperative and postoperative assessments for comparison, and the test is not limited to a particular aspect of cognitive function, while its content should include memory for what is heard, memory for visual content, and the ability to processing and executive abilities in these three areas (Collie et al., 2002). The diagnosis of POCD in this study was made using the neuropsychological test scale set suggested by the Canadian research group.

In the cognitive function tests of patients in the three groups, it was found that the test scores of patients in Group Mo decreased compared with the preoperative test scores on the Hopkins Verbal Learning Test (HVLT), Trail Making Test (TMT) and the Stroop. Color Word Interference Test (SCWIT) at 5 and 30 days after surgery, and the test scores at 5 and 30 days after surgery decreased significantly compared with those in the Group N and Group Mi. The difference was statistically significant, suggesting that the patients with moderate anemia had a decrease in memory, attention, calculation and recall ability after surgery, while those abilities were not affected in the patients without or with mild anemia after surgery.

The results of this study suggest that the incidence of POCD in Group N was lower than that obtained from other studies which is between 8.9 and 46.1%, indicating that the influence of other factors on the results of this experiment was taken into account as much as possible in the design of the experiment and was kept to a low level. The results of this study showed that patients in the Group Mi were less likely to have postoperative cognitive impairment than those with anemia, and that patients in the Mo group showed a decline in cognitive function at postoperatively 5th and 30th days. This suggests a positive correlation between the degree of anemia and the incidence of POCD. Previous studies have found that patients with chronic hemorrhagic anemia are more likely to get infections (Parvizi et al., 2008). This may be related to chronic blood loss and prolonged loss of albumin and globulin, resulting in decreased protein and immune deficiency in patients with hypoproteinemia. When the patient’s immune function is impaired, the lymphatic immune factor of the cells begins to decline, the secretion of immunoglobulin decreases, the phagocytosis of leukocytes is affected, and the activity of complement C3 and C4 in the serum also decreases, which makes the patient more vulnerable to external bacterial and viral infections (Tang et al., 2016; Chen, 2017; Tao et al., 2018; Xiong et al., 2019). In patients with anemia, the level of inflammatory factors in the peripheral blood will increase continuously when the infection occurs and leads to acute inflammation, so inflammatory factors can also be used as a reference indicator to determine the occurrence of infection at an early stage (Chen et al., 2021).

In patients with POCD in this study, the serum levels of inflammatory factors at different time points after surgery were found to be increased to different degrees compared with those before surgery, and the increase was statistically significant, which is consistent with the results obtained in previous studies.

It has been suggested in the literature that the occurrence of POCD may be related to postoperative peripheral and central neuroinflammation, and when the trauma-induced inflammation is not properly regulated, the persistent neuroinflammation may interfere with synaptic functions related to cognition and learning and memory (Saxena And Maze, 2018). Therefore, an in-depth study of the influence of neuroinflammation in POCD is important for further exploring the mechanisms of POCD and for the prevention and treatment of POCD in clinical practice. Under normal physiological conditions, the levels of inflammatory factors in plasma are maintained at low levels, while the expression of inflammatory factors increases when the body is subjected to intense external injuries or when the body is in certain disease states (Shen, 2013; Mattavelli et al., 2015; Huang, 2016; Zhang et al., 2016).

The expression of IL-6, a pro-inflammatory factor produced by T lymphocytes, is strictly regulated by the transcriptional machinery, but when the body is traumatized and immune function is compromised, the transcriptional machinery is deregulated and the transcription factor NF-κB is activated, leading to increased synthesis and release of inflammatory factors (HU et al., 2019), and IL-6’s expression continues to increase under the uncontrolled transcriptional machinery, which has pathological effects on the body. The level of IL-6 is positively correlated with the degree of trauma to the body. In an injured organism, endothelial cells and phagocytes release inflammatory factors in response to stress, and inflammatory factors such as TNF-α and IL-1, which are the initiators of inflammation, can trigger a cascade of inflammation by promoting the entry of leukocytes into the bloodstream, and when the levels of TNF-α and IL-1 increase, the levels of IL-6 increase as well (Izquierdo et al., 2003; Michetti et al., 2016).

Inflammation can disrupt the blood-brain barrier (BBB). The inflammatory factor TNF-α activates NF-κB signaling pathway, which increases prostaglandin E synthesis through upregulation of COX-2 and activation and dysfunction of endothelial cells in the blood-brain barrier, thereby increasing the permeability of the blood-brain barrier, while other inflammatory factors, such as IL-1, also use signaling pathway to increase the permeability of the blood-brain barrier by converging on other signaling pathways that upregulate COX-2. Other inflammatory factors such as IL-1 can also increase the permeability of blood-brain barrier by upregulating other signaling pathways of COX2. Currently, increased blood-brain barrier permeability is recognized as the initial event in the development of neuroinflammation. All types of inflammatory responses, including infectious and non-infectious inflammatory responses, can lead to cognitive impairment, and peripheral inflammatory responses can trigger central inflammatory responses through multiple pathways. While inflammatory factors at normal physiological levels protect nerve cells in the brain and promote repair of damaged nerve cells. When a central inflammatory response occurs, inflammatory factors are increased, and they contribute to cognitive impairment through a variety of mechanisms, including interference with nerve cell function, inhibition of neuronal regeneration in the hippocampus, and induction of apoptosis. When inflammatory responses in the CNS act on synapses, their connectivity is impaired, which in turn affects the nervous system and leads to neurological deficits. The inflammatory factors IL-6, IL-1, S-100β protein, and TNF-α play an important role in POCD, and Konsman have found that inflammatory factors such as IL-1β, IL-6, and TNF-α are involved in the reduction of neuronal regeneration due to inflammation.

In conclusion, women with moderate anemia were more likely to experience cognitive dysfunction after uterine fibroid surgery than those with mild anemia, particularly in the aspects of orientation, memory, attention, and computational skills. In patients with chronic anemia, the incidence of infections and inflammatory reactions is increased because of protein loss and decreased immune function. We consider that the decline in cognitive function in patients with POCD in this study may be related to the inflammatory response caused by chronic preoperative anemia and the central nervous system damage mediated by the inflammatory response caused by surgery. Therefore, attention should be paid to patients with uterine fibroids who have chronic anemia in combination with surgery, and if necessary, we should actively intervene to improve the symptoms of anemia by supplementing iron and increasing the level of hemoglobin to reduce the risk of infection and inflammation, thus reducing the incidence of POCD and preventing cognitive impairment in patients after surgery.

## Data Availability

The raw data supporting the conclusion of this article will be made available by the authors, without undue reservation.
